# Factitious Urticaria

**Published:** 1890-03

**Authors:** Alfred J. Harrison

**Affiliations:** Physician to the Bristol General Hospital


### FACTITIOUS URTICARIA. By ALFRED J. HARRISON, M.B.(Lond.), Physician to the Bristol General Hospital.

The subject of "Factitious Urticaria" is a remarkable one, whether looked at from a dermatological or a physiological point of view, or from its comparative rarity. That we should meet with persons who have a skin so sensitive as to resent the slightest irritation, appears, perhaps, more surprising than that the gastric mucous membrane should occasionally be subject to intense excitement at the presence of various articles of food. We seem to expect the skin to resist pressure and irritation as one of its functions, and are therefore surprised when the phenomena of factitious urticaria occur.

There is no doubt that persons are more prone to it in the adolescent period than any other; for it is a varying affection, and I should say that cases happen much more frequently amongst males than females. I have met with cases amongst people at the middle period of life; but I do not recollect a case over fifty years of age. It does not follow that because a person becomes subject to it, that he should continue to be so. It may be long in disappearing; and time, more than drugs, is the curative agent.

Indigestion may be a coincidence, but I doubt whether it has any other connection. The two following cases are very typical, and the subjects were brothers:

E. G. was 19 years old when I saw him first, at the Bristol General Hospital in March, 1887. He was a compositor by trade, had plenty of muscular strength and a fairly florid complexion. He liked an active life, and thought his work was too confining. He complained of a great deal of irritation of the skin. The slightest blow would be followed by a very distinct "whealing;" and if he used a towel, at all rough, to wipe his face, arms, or body, after washing, his skin would become very red. These symptoms have only come on during the last few months, and he thought they arose from his more sedentary occupation. He made no marked complaint of suffering from indigestion or any other ailment.

Having seen several cases of "factitious urticaria," I examined his skin, and made the following experiments: I took the smooth end of a penholder, and printed the word "Edward" on the lower scapular region, and then made a few plain crosses on his arms and shoulders. The following phenomena occurred: Almost immediately the letters and markings appeared as thin red lines; then the redness extended over the surrounding skin, forming a pink suffusion. In another minute or two the red lines of the letters began to turn pale, and gradually most distinct white wheals—the exact outlines of the letters and marks—rose up prominently above the now deeper suffused surface, and became so distinct and discrete that a blind person could have made them out as readily as raised type on a page. Next, the surrounding redness gradually became paler, until at last there were only the raised wheals left, and these slowly disappeared. A slight itching of the skin of the part was felt, and this was apparently the only subjective symptom.

The other brother, W. G., æt. 14, a clerk, had symptoms very much resembling his brother, but in addition had been liable for several years to attacks of epistaxis; and if he cut his finger, it bled very freely; and if he engaged in any game, such as football or cricket, where he was liable to be pushed or hit with a ball, the parts affected would swell very quickly, and become very red—so much so that, although liking games very much, and being a fairly strong boy for his years, he was inclined to give them up.

With both of them the wheals were more easily produced in hot than in cold weather; and they would occur on any parts of the body or the extremities, except the palms, the soles, and the head. The explanation of the phenomena would appear to be this: First of all, the marking of the skin sets up an irritation, which is conveyed to the capillaries of the part through the vaso-motor system; the capillaries dilate, causing the red lines to appear; then the adjoining surfaces participate in the action, and the suffusion spreads from the parts first irritated. Shortly, the capillaries first affected, begin to get paralysed, white distinct wheals appear, and serum is forced out. I think there can be no question about that, because the wheals pit distinctly upon pressure. The surrounding blood-vessels dilate more and more, and the redness deepens and increases. By-and-by, the capillaries begin to contract again, the suffusion gradually disappears, and, last of all, the serum of the wheals is re-absorbed.

I have seen many such cases. One man was engaged at a tobacco-factory, and he showed very marked symptoms.

I have treated these cases with tonics, astringents, mild aperients, arsenic, belladonna, bromides, ergot, and salicylate of soda, the extract of nux vomica, strychnia; or two or more of the remedies in combination. Some of them seem to exert very little curative action. Ergot gave the best results; but, at the best, drugs seem to be rather inoperative.

Cases of factitious urticaria should also be looked at from a medico-legal aspect, and the consideration is very important.

Take the case of the younger boy, W. G. Suppose a school-teacher gave him a few smart strokes with a cane on the back or arms. The appearance of the back, a short time afterwards, would be quite out of proportion to the severity of the punishment, and a very prejudiced opinion, so far as the master was concerned, might very readily be formed, and a charge of undue violence be very easily set up.

As an interesting sequel to the cases of the two brothers, I may state that since they were under my care, they have both entered the Navy, and have developed into two as fine and healthy-looking young fellows as could be met with, and the "urticarial" phenomena are very much diminished.

